# Supplementary material for: A Novel Jeilongvirus from Florida, USA, Has a Broad Host Cell Tropism Including Human and Non-Human Primate Cells
Source: Pathogens. 2024 Sep 26;13(10):831. doi: 10.3390/pathogens13100831 (PMC11510135; doi:10.3390/pathogens13100831)
Supplement: Supplementary file 1 [file pathogens-13-00831-s001.zip › Supplemental Figure S1.pdf]

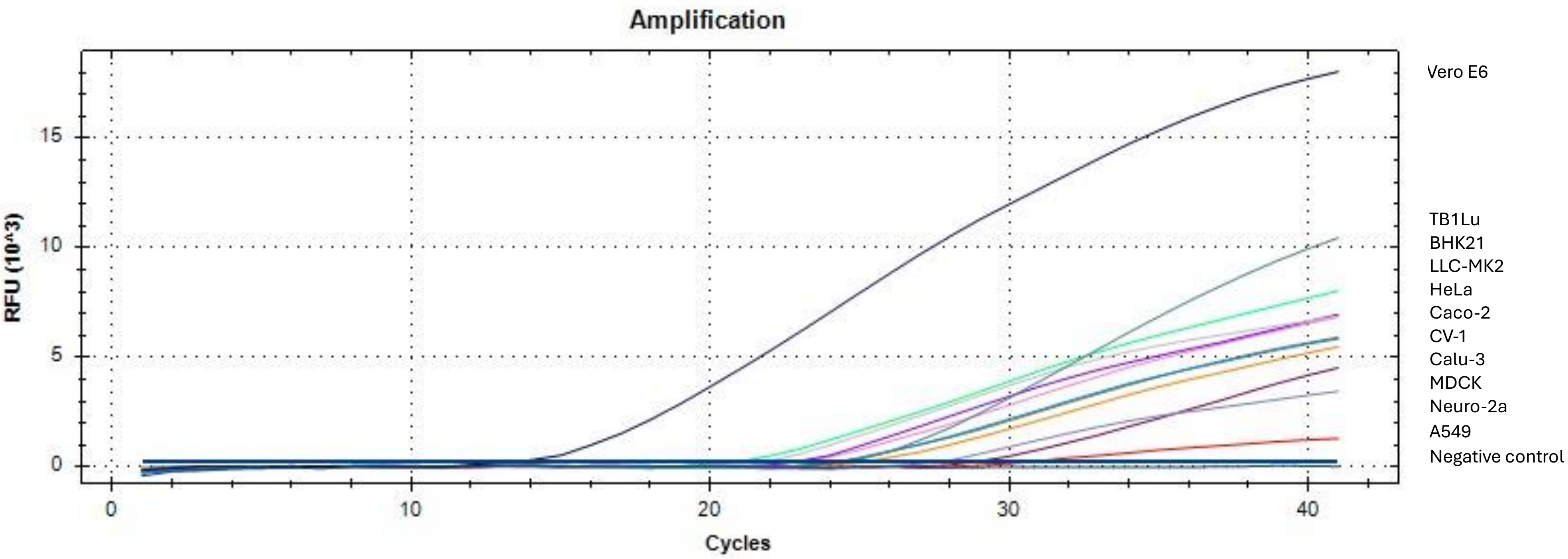

Figure S1: rRT-PCR curve of cell lines inoculated with GRJV1 and negative controls maintained in parallel
