# Supplementary material for: A Novel Jeilongvirus from Florida, USA, Has a Broad Host Cell Tropism Including Human and Non-Human Primate Cells
Source: Pathogens. 2024 Sep 26;13(10):831. doi: 10.3390/pathogens13100831 (PMC11510135; doi:10.3390/pathogens13100831)
Supplement: Supplementary file 1 [file pathogens-13-00831-s001.zip › Supplemental Figure S2.pdf]

	Apodemus agrarius jeilongvirus	Jingmen Apodemus agrarius jeilongvirus 1	Jingmen rodent jeilongvirus 1	Wenzhou Apodemus agrarius jeilongvirus 1	Paju Apodemus paramyxovirus 1	Apodemus draco jeilongvirus	Wufeng Apodemus chevrieri jeilongvirus 1	Wufeng rodent jeilongvirus 4	Beilong virus	Rattus tanezumi jeilongvirus	Wenzhou Rattus norvegicus jeilongvirus 1	Wufeng Rattus nitidus jeilongvirus 1	Longquan Niviventer fulvescens jeilongvirus 1	Longquan Niviventer fulvescens jeilongvirus 2	Rodent paramyxovirus	Eothenomys eva jeilongvirus	Ninove microtus virus	Wufeng Eothenomys melanogaster jeilongvirus 1	Pohorje myodes paramyxovirus 1	Jingmen Apodemus agrarius jeilongvirus 2	Jun jeilongvirus	Mus musculus jeilongvirus	J virus	Meliandou mastomys virus	Meliandou praomys virus	Mount Mabu Lophuromys virus 2	Gainesville rodent jeilongvirus 1	Jingmen rodent jeilongvirus 2	Paju Apodemus paramyxovirus 2	Meliandou lophuromys virus	Mount Mabu Lophuromys virus 1	Wufeng Typhlomys cinereus jeilongvirus 1	Feline paramyxovirus 163	Belerina virus	Jingmen bat jeilongvirus 7	Wenzhou bat jeilongvirus 1	Bat paramyxovirus 16797	Bat paramyxovirus 17770	Shaan virus	Longquan Niviventer niviventer jeilongvirus 1	Wenzhou rodent jeilongvirus 1	Niviventer confucianus jeilongvirus	Wenzhou Rattus losea jeilongvirus 2	Wenzhou rodent jeilongvirus 2	Memana virus	Ruloma virus	Wufeng rodent jeilongvirus 6	Ochotona cansus jeilongvirus	Bank vole virus 1
Apodemus agrarius jeilongvirus	100.0																																																
Jingmen Apodemus agrarius jeilongvirus 1	99.8	100.0																																															
Jingmen rodent jeilongvirus 1	99.4	99.4	100.0																																													
Wenzhou Apodemus agrarius jeilongvirus 1	98.9	98.9	98.7	100.0																																												
Paju Apodemus paramyxovirus 1	98.9	99.0	98.8	98.6	100.0																																											
Apodemus draco jeilongvirus	82.7	82.8	82.8	82.4	82.8	100.0																																										
Wufeng Apodemus chevrieri jeilongvirus 1	83.2	83.2	83.3	83.0	83.2	87.2	100.0																																									
Wufeng rodent jeilongvirus 4	83.2	83.2	83.2	83.1	83.1	87.1	99.9	100.0																																								
Beilong virus	77.4	77.4	77.4	77.2	77.4	77.5	77.9	77.9	100.0																																							
Rattus tanezumi jeilongvirus	77.8	77.9	77.7	77.4	77.7	77.2	77.4	77.4	88.9	100.0																																						
Wenzhou Rattus norvegicus jeilongvirus 1	78.1	78.1	77.9	77.8	77.9	77.1	77.6	77.6	89.9	90.9	100.0																																					
Wufeng Rattus nitidus jeilongvirus 1	77.4	77.5	77.3	77.3	77.6	77.8	77.3	77.1	82.7	83.2	83.8	100.0																																				
Longquan Niviventer fulvescens jeilongvirus 1	77.1	77.2	77.1	77.3	77.1	76.7	77.0	77.0	80.7	79.9	80.2	80.0	100.0																																			
Longquan Niviventer fulvescens jeilongvirus 2	77.6	77.6	77.3	77.6	77.7	76.9	77.3	77.3	79.9	79.2	79.7	79.3	85.5	100.0																																		
Rodent paramyxovirus	78.5	78.5	78.8	78.4	78.7	77.9	78.0	78.0	79.2	79.3	79.1	79.2	78.7	78.1	100.0																																	
Eothenomys eva jeilongvirus	74.2	74.2	74.3	74.0	74.3	74.8	73.7	73.7	74.2	73.5	74.0	73.6	73.5	73.8	74.6	100.0																																
Ninove microtus virus	75.6	75.8	75.5	75.3	75.8	74.1	74.5	74.5	74.9	75.2	75.3	74.9	75.5	74.8	75.2	75.7	100.0																															
Wufeng Eothenomys melanogaster jeilongvirus 1	74.0	73.9	73.9	73.6	73.9	73.8	73.7	73.7	74.1	73.9	74.0	74.3	73.6	73.8	74.4	75.2	78.8	100.0																														
Pohorje myodes paramyxovirus 1	73.8	73.7	73.7	73.5	73.7	73.6	73.9	73.9	74.1	74.1	74.2	74.2	74.2	74.3	75.1	75.1	78.5	77.1	100.0																													
Jingmen Apodemus agrarius jeilongvirus 2	74.9	74.9	74.6	74.4	74.6	74.4	74.0	74.1	74.5	75.0	74.5	75.0	73.6	74.5	75.3	72.4	72.4	72.1	72.4	100.0																												
Jun jeilongvirus	74.7	74.8	74.6	74.4	74.6	74.3	73.9	73.9	74.4	75.1	74.5	75.0	73.6	74.5	75.5	72.4	72.4	72.1	72.5	99.8	100.0																											
Mus musculus jeilongvirus	74.9	74.9	74.6	74.4	74.6	74.3	73.9	74.1	74.4	75.1	74.5	75.0	73.6	74.5	75.3	72.4	72.4	72.1	72.5	99.8	99.8	100.0																										
J virus	74.4	74.4	74.4	73.9	74.1	74.0	73.9	74.0	74.0	74.7	74.0	74.3	73.2	74.4	74.5	72.3	71.9	71.7	71.7	94.4	94.3	94.3	100.0																									
Meliandou mastomys virus	69.9	70.0	70.2	69.9	69.8	69.9	69.3	69.4	69.4	70.4	69.6	69.9	69.3	69.8	70.7	68.9	68.9	69.0	68.0	69.9	69.9	69.9	69.8	100.0																								
Meliandou praomys virus	70.4	70.3	70.3	69.9	70.2	69.5	69.5	69.5	69.8	69.5	69.6	69.8	69.9	70.1	70.2	68.5	69.0	69.5	68.5	69.1	69.0	69.1	69.0	78.4	100.0																							
Mount Mabu Lophuromys virus 2	70.3	70.3	70.2	70.2	70.3	70.0	70.3	70.3	69.5	69.3	69.4	69.7	68.7	68.7	69.7	68.8	69.0	70.7	69.3	69.5	69.6	69.6	69.9	74.0	72.6	100.0																						
Gainesville rodent jeilongvirus 1	68.5	68.6	68.8	68.3	68.6	67.9	68.1	68.1	67.9	67.9	67.9	68.0	67.6	68.4	68.8	67.6	67.4	67.3	67.4	67.2	67.4	67.3	67.6	69.6	68.7	68.0	100.0																						
Jingmen rodent jeilongvirus 2	62.5	62.5	62.6	62.4	62.5	61.4	61.7	61.8	61.7	61.6	61.4	61.7	61.5	62.5	61.7	60.9	62.2	62.4	61.4	61.5	61.5	61.5	61.3	63.8	63.7	62.1	62.2	100.0																					
Paju Apodemus paramyxovirus 2	62.4	62.5	62.5	62.2	62.3	61.6	61.8	61.8	61.3	61.5	61.4	62.0	61.7	62.2	61.8	60.6	62.5	61.9	61.4	61.7	61.7	61.7	61.4	63.6	63.9	62.5	62.0	90.1	100.0																				
Meliandou lophuromys virus	61.3	61.3	61.4	61.1	61.5	61.4	61.3	61.3	61.3	60.8	61.0	61.1	60.2	61.4	61.4	60.5	61.6	62.2	61.2	62.0	62.1	62.0	61.8	63.8	63.8	62.4	62.7	79.0	78.6	100.0																			
Mount Mabu Lophuromys virus 1	61.4	61.5	61.5	61.4	61.3	61.1	61.2	61.2	61.1	61.8	61.3	61.4	61.0	61.6	61.9	61.4	62.7	61.6	61.5	62.1	62.2	62.2	62.4	63.9	63.9	62.9	62.1	80.2	79.8	85.8	100.0																		
Wufeng Typhlomys cinereus jeilongvirus 1	59.2	59.3	59.2	59.2	59.1	60.4	59.3	59.3	59.4	59.3	59.3	59.7	60.6	60.4	59.9	59.1	60.4	60.3	60.7	60.7	60.7	60.8	60.6	61.0	60.7	59.7	59.6	63.4	63.6	63.4	63.8	100.0																	
Feline paramyxovirus 163	61.7	61.7	61.9	61.7	61.7	61.4	61.5	61.5	60.2	60.3	59.7	60.1	60.6	61.0	61.3	61.0	60.3	60.3	61.5	60.9	60.9	60.8	60.4	61.1	61.1	61.9	60.2	63.5	63.9	63.0	63.4	61.0	100.0																
Belerina virus	57.9	57.8	57.9	57.8	57.9	56.9	57.6	57.6	58.1	58.3	58.5	57.6	58.4	58.8	57.4	57.4	57.3	57.5	58.3	58.5	58.6	58.5	58.2	58.6	58.2	56.8	58.3	60.8	60.4	59.5	60.1	58.0	58.3	100.0															
Jingmen bat jeilongvirus 7	58.1	58.1	58.3	58.1	58.0	57.9	58.1	58.0	56.8	57.7	57.5	57.9	58.1	57.5	58.3	57.4	58.1	57.8	58.5	58.8	59.1	58.8	58.4	60.3	58.7	58.2	57.6	62.1	62.2	61.2	61.7	58.4	59.5	62.5	100.0														
Wenzhou bat jeilongvirus 1	58.6	58.6	58.6	58.6	58.5	58.4	58.5	58.5	57.8	57.3	57.8	58.1	57.7	57.8	57.6	58.0	59.0	57.9	58.1	58.3	58.4	58.3	57.5	60.1	58.6	58.2	58.6	61.9	61.7	61.3	61.7	58.4	60.8	62.4	68.1	100.0													
Bat paramyxovirus 16797	60.1	60.1	60.2	60.0	60.1	59.6	59.6	59.6	59.3	59.0	58.4	59.2	58.8	59.2	59.0	58.7	59.3	59.5	58.4	59.9	59.8	59.8	59.7	59.7	59.5	60.6	58.4	61.2	61.7	61.1	61.1	60.1	61.4	58.9	59.9	59.7	100.0												
Bat paramyxovirus 17770	59.2	59.2	59.3	59.1	59.3	59.1	58.5	58.5	59.3	58.8	58.8	59.2	59.2	58.9	58.5	58.8	59.5	59.3	58.4	58.9	59.0	59.0	58.9	59.1	59.3	60.0	58.5	61.5	60.9	61.0	61.7	59.0	60.4	58.7	59.4	59.0	87.2	100.0											
Shaan virus	59.8	59.8	59.9	59.8	60.1	59.0	59.3	59.3	59.2	59.3	59.2	59.0	58.6	58.4	59.0	58.8	59.2	59.4	59.1	59.3	59.4	59.3	58.7	60.1	59.7	60.0	58.9	61.0	60.7	60.3	60.5	59.2	61.3	58.9	59.4	59.7	78.5	78.2	100.0										
Longquan Niviventer niviventer jeilongvirus 1	58.0	58.0	57.7	57.7	57.5	57.4	56.5	56.5	56.8	57.0	56.9	56.7	56.8	57.3	57.4	55.8	57.2	56.3	57.7	56.9	57.1	57.0	56.6	57.5	57.5	57.2	57.5	58.6	58.5	59.1	58.3	57.7	57.1	56.5	56.8	57.1	57.1	56.8	55.9	100.0									
Wenzhou rodent jeilongvirus 1	58.3	58.3	57.9	57.9	57.8	57.6	56.7	56.7	57.1	57.2	57.1	56.8	57.1	57.5	57.6	56.1	57.5	56.6	57.6	57.2	57.4	57.3	56.8	57.4	57.7	57.5	57.8	58.6	58.6	59.3	58.5	57.9	57.1	56.5	57.1	57.2	57.3	57.0	56.1	99.2	100.0								
Niviventer confucianus jeilongvirus	58.1	58.1	58.1	58.2	58.0	57.6	57.1	57.1	56.9	57.4	57.1	56.8	57.2	57.0	57.3	56.2	57.9	56.7	57.7	56.7	56.8	56.8	56.6	57.7	57.8	56.8	57.8	58.5	58.8	59.1	59.2	57.7	57.1	56.2	57.3	57.4	56.8	56.2	55.6	93.9	94.3	100.0							
Wenzhou Rattus losea jeilongvirus 2	58.0	57.9	57.8	57.9	58.1	58.2	57.7	57.7	57.3	58.3	58.1	58.3	57.5	57.5	57.8	57.8	58.1	57.7	58.6	58.1	58.2	58.2	57.6	58.2	57.9	57.5	58.7	58.3	58.2	59.5	58.8	58.1	57.9	55.5	57.3	56.5	57.7	56.7	56.5	76.0	75.9	76.0	100.0						
Wenzhou rodent jeilongvirus 2	58.3	58.2	58.1	58.2	58.3	58.5	58.3	58.3	57.3	58.4	58.4	58.5	57.4	57.8	58.0	57.9	58.2	57.8	58.5	58.5	58.5	58.5	57.9	58.6	58.2	58.0	58.7	58.1	58.2	59.5	59.0	58.1	57.6	55.3	57.2	56.5	57.5	57.1	56.8	75.9	76.0	76.1	98.4	100.0					
Memana virus	57.8	57.8	57.7	57.5	57.7	57.0	57.5	57.5	57.3	57.4	57.6	57.5	57.6	57.4	56.9	56.7	58.3	56.8	58.4	57.0	57.1	57.1	56.8	58.3	57.9	57.2	58.3	58.3	58.8	59.8	60.9	58.8	57.8	56.4	58.4	56.8	57.3	56.6	56.8	72.1	72.3	72.4	74.5	74.6	100.0				
Ruloma virus	58.0	58.0	58.1	57.9	58.1	56.6	57.0	57.0	56.8	57.2	57.0	55.7	56.7	56.7	56.6	57.2	57.5	56.4	56.6	56.4	56.5	56.4	56.0	57.3	57.2	56.3	57.9	57.8	57.9	57.5	58.5	57.7	57.3	55.3	57.3	56.0	57.6	57.0	57.2	64.5	64.7	64.4	65.6	65.9	65.5	100.0			
Wufeng rodent jeilongvirus 6	58.7	58.7	58.6	58.7	58.8	58.3	58.4	58.1	57.1	57.0	57.4	57.2	56.9	58.2	57.4	56.6	58.0	56.9	57.1	57.3	57.3	57.2	57.4	59.6	59.5	59.2	57.7	58.4	57.5	57.8	57.6	58.5	57.8	56.2	55.6	57.2	57.4	56.8	57.0	60.0	60.1	60.1	60.5	60.6	60.6	60.4	100.0		
Ochotona cansus jeilongvirus	54.7	54.7	54.6	54.7	54.6	54.5	54.4	54.4	54.6	54.8	54.4	54.1	55.5	55.2	53.5	53.8	54.8	54.7	54.4	54.4	54.7	54.4	53.9	54.9	54.4	54.8	54.3	55.3	54.8	56.1	56.0	55.1	54.9	53.9	53.9	54.2	54.4	54.8	53.8	57.1	57.2	57.9	58.3	58.5	58.5	57.4	58.3	100.0	
Bank vole virus 1	51.1	50.9	50.9	50.9	51.3	50.0	50.7	50.7	50.3	49.8	49.9	50.8	51.0	51.1	50.6	50.5	50.9	50.6	51.4	50.5	50.5	50.5	51.5	49.3	50.7	50.2	49.7	52.8	52.3	52.4	52.4	51.1	52.3	49.7	50.5	49.5	51.0	51.2	50.0	49.6	49.6	49.2	51.4	51.1	49.3	50.0	50.0	50.3	100.0

Figure S2: Sequence identity matrix based on the amino acid sequence of the L protein of GRJV1 (highlighted in red) compared to 47 other *jeilongviruses* and bank vole virus 1
